# Comprehensive geriatric assessment predicts radiation-induced acute toxicity in prostate cancer patients

**DOI:** 10.1007/s00066-023-02132-3

**Published:** 2023-09-02

**Authors:** Katarzyna Paal, Bettina Stranz, Eva-Maria Thurner, Uwe Langsenlehner, Wilfried Renner, Thomas Baptist Brunner, Tanja Langsenlehner

**Affiliations:** 1 Department of Therapeutic Radiology and Oncology, Comprehensive Cancer Center, Medical University of Graz, 8036 Graz, Austria; 2 Outpatient Center for Internal Medicine, PRODOCplus, 8020 Graz, Austria; 3 Clinical Institute of Medical and Chemical Laboratory Diagnostics, Medical University of Graz, 8036 Graz, Austria

**Keywords:** Prostate neoplasms, Radiation therapy, Elderly patients, Adverse effects, Predictive factors, Personalized medicine, Geriatric oncology

## Abstract

**Purpose:**

The purpose of the present prospective study was to evaluate the significance of geriatric conditions measured by a comprehensive geriatric assessment (GA) for the prediction of the risk of high-grade acute radiation-induced toxicity.

**Methods:**

A total of 314 prostate cancer patients (age ≥ 65 years) undergoing definitive radiotherapy at a tertiary academic center were included. Prior to treatment, patients underwent a GA. High-grade toxicity was defined as acute toxicity grade ≥ 2 according to standard RTOG/EORTC criteria. To analyze the predictive value of the GA, univariable and multivariable logistic regression models were applied.

**Results:**

A total of 40 patients (12.7%) developed acute toxicity grade ≥ 2; high grade genitourinary was found in 37 patients (11.8%) and rectal toxicity in 8 patients (2.5%), respectively. Multivariable analysis revealed a significant association of comorbidities with overall toxicity grade ≥ 2 (odds ratio [OR] 2.633, 95% confidence interval [CI] 1.260–5.502; *p* = 0.010) as well as with high-grade genitourinary and rectal toxicity (OR 2.169, 95%CI1.017–4.625; *p* = 0.045 and OR 7.220, 95%CI 1.227–42.473; *p* = 0.029, respectively). Furthermore, the Activities of Daily Living score (OR 0.054, 95%CI 0.004–0.651; *p* = 0.022), social status (OR 0.159, 95%CI 0.028–0.891; *p* = 0.036), and polypharmacy (OR 4.618, 95%CI 1.045–20.405; *p* = 0.044) were identified as independent predictors of rectal toxicity grade ≥ 2.

**Conclusion:**

Geriatric conditions seem to be predictive of the development of high-grade radiation-induced toxicity in prostate cancer patients treated with definitive radiotherapy.

## Introduction

Prostate cancer (PCa) is predominantly a disease of older adults with a median age at diagnosis of 68 years [1]. With the exponential aging of the population and increasing life expectancy, especially in developed countries, the burden due to prostate cancer is expected to increase substantially in the future.

External beam radiotherapy represents a highly efficacious treatment modality for prostate cancer and offers a particular advantage in patients who are unsuitable for surgery because of comorbidity or evidence of extraprostatic extension of the cancer. However, management of PCa is often challenging because, even without treatment, disease progression can occur slowly in many patients but it has also been demonstrated that death as a result of PCa increases with age, despite increasing death rates from competing causes [2].

Life-expectancy is a major determinant of the potential to benefit from curative therapy. In prostate cancer, predicted life expectancy has directly been incorporated into treatment guidelines [3]. However, life expectancy varies substantially between individuals within a given age group [4]. Thus, advanced age alone should not preclude effective treatment for prostate cancer but it is necessary to assess the risks and benefits of treatment in elderly patients to avoid interventions that might decrease health-related quality of life without prolonging survival.

The geriatric assessment (GA) is defined as a multidimensional diagnostic process, focusing on determining an older person’s medical, psychosocial, and functional capabilities to objectively appraise the health status of older people in order to develop a coordinated and integrated plan for treatment and long-term follow-up [5]. GA-guided treatment plans have been shown to improve overall survival (OS), quality of life, physical function, and decrease the risk of hospitalization [6, 7]. The available data also support the value of GA as an effective tool to predict the patient’s tolerance of cytotoxic interventions [8]. Most previous studies focused on the prediction of chemotherapy toxicity and showed that the factors most consistently associated with toxicity were functional status and comorbidity [9–14]. Other identified risk factors were cognitive deficiencies, lack of social support, poor mood status, falls, and nutritional status [9, 15]. Other publications also showed a correlation between impairments measured with GA tools and risk of premature termination of cancer treatment [13, 16–19].

Geriatric conditions are also likely to influence analogous facets of radiation treatment, specifically the patient’s ability to complete the intended radiation treatment duration and the ability to tolerate radiotherapy-related side-effects. Thus, implementation of GA might be an effective tool for the identification of older adults who are at high risk of radiotherapy complications. However, to date, little is known about the ability of a GA to predict toxicity in elderly cancer patients undergoing curative radiotherapy [20–23].

The aim of the present study was to identify geriatric conditions measured by GA that are predictive of the development of high-grade radiation-induced toxicities in a cohort of prostate cancer patients treated with definitive radiotherapy.

## Materials and methods

This single-center prospective cohort study was performed including 314 prostate cancer patients treated at the tertiary academic center.

Patients were eligible for inclusion if aged 65 years or above, candidates to definitive radiation treatment and provided written informed consent. At inclusion in the cohort, all study participants completed a routine clinical questionnaire including family history, medication, previous surgery, comorbidities, and smoking habits.

Before initiation of the radiotherapy, a comprehensive GA was completed. The measures included in the GA were chosen for their reliability, validity, brevity, and prognostic ability to determine risk for morbidity or mortality in older patients. The GA tools are summarized in Table 1 and comprised a health care provider and a patient portion. The health care provider portion consisted of two items: Timed Up and Go Test (TUG) and the Mini-Mental State Examination (MMSE). The patient portion consisted of self-reported measures of functional status, nutrition, social support/function, mood, comorbidity and medications. A member of the radiation oncologist team assisted those who needed help with completing the questionnaires; the assessments were performed under the supervision of a geriatric oncologist.

**Table 1:** Health domains measured with geriatric assessment tools

Domain	Measure	Number of items	Description	Range of scores
Functional status	Activities of Daily Living (ADL) [45]	10	Measures limitations in a wide range of physical functions	0–100 (higher score: better physical function)
	Instrumental Activities of Daily Living (IADL) [46]	8	Measures ability to complete activities required to maintain independence in the community	0–8 (higher score: less need for assistance)
	Timed Up and Go Test (TUG) [47]	2	Measures how many seconds it takes to stand up from an armchair, walk a distance of 3 m, turn, walk back, sit down again	Time > 0 (higher score: higher risk of fall)
Nutritional status	Mini Nutritional Assessment (MNA) [48]	18	Assesses nutritional status in elderly patients	0–30 (higher score: better nutritional status)
Social status and support	Social status and support Survey (SOS) [49]	4	Measures the perceived availability of social support and level of social activity	0–25 (higher score: higher social support, higher social activity and better conditions)
Cognition	Mini-Mental State Examination (MMSE) [50]	11	Provides a quantitative assessment of cognitive impairment	0–30 (higher score: better cognitive state)
Mood	Mini-Geriatric Depression Scale (mini-GDS) [51]	15	Assesses the level of depression and anxiety	0–15 (lower score: better mood state)
Co-morbidities	Charlson Comorbidity Index (CCI) [52]	–	Presence/absence of 19 comorbid illnesses: number of comorbid illnesses	0–38 (higher score: higher comorbidity burden)
Polypharmacy	Number of medications	–	Assesses the number of currently prescribed medications	≥ 0 (higher score: higher number of medications)

Image-guided radiotherapy with high energy photons was generally performed using volumetric intensity-modulated arc therapy (VMAT) techniques to encompass the prostate and seminal vesicles. The total dose, prescribed to the International Commission on Radiation Units and Measurement point, ranged from 74–78 Gy delivered in 2 Gy per fraction dependent on risk situation. Hypofractionated radiotherapy with a total dose of 60 Gy (3 Gy per single fraction) was delivered in 13 patients. Treatment was performed daily, 5 days/week.

In patients with low risk disease, the clinical target volume (CTV) encompassed the prostate. In intermediate and high-risk disease, the CTV included the prostate and 75% of the seminal vesicles (SV), in case of SV involvement, the entire seminal vesicles were included. The planning target volume (PTV) was defined as the CTV with a margin of 7 mm in all dimensions, except for the posterior aspect (prostate–rectal interface), where the margin was 5 mm. Pelvic lymph node irradiation was performed in 7 patients with clinical lymph node involvement.

The rectum was segmented from above the anal verge to the turn into the sigmoid colon, including the rectal contents. The dose–volume constraints were defined according to the Quantitative Analysis of Normal Tissue Effects in the Clinic (QUANTEC) recommendations [24, 25]. In patients treated with conventionally fractionated radiotherapy, the following dose constraints had to be fulfilled: V50 < 50%, V60 < 35%, V65 < 25%, V70 < 20%, and V75 < 10%. The urinary bladder was outlined as entire organ. The bladder dose constraints to be fulfilled were as follows: V50 < 50%, V60 < 40%, V70 < 25%.

The dose constraints for the rectum and bladder in patients treated with hypofractionated radiotherapy were derived from the CHHIP trial [26].

Acute genitourinary and gastrointestinal toxicity was regularly assessed during the radiation therapy course and graded according to standard European Organisation for Research and Treatment of Cancer (EORTC)/Radiation Therapy Oncology Group (RTOG) criteria.

The study complied with the Declaration of Helsinki and was performed according to the national law. The protocol has been approved by the local Ethical Committee (approval number: EK 31-437 ex 18/19). Written informed consent was obtained from all participants.

The primary endpoint was the development of high-grade acute genitourinary or rectal toxicity that was defined as acute toxicity grade ≥ 2 according to standard RTOG/EORTC criteria. Comparison of groups was done using student’s t‑test, rank sum test, χ^2^ test, and analysis of variance (ANOVA). The relationship between GA results and age was studied by nonparametric tests. The association of GA variables with acute toxicity was assessed using univariable logistic regression analysis. The variables that reached a *p*-value of less than 0.1 were further examined in a multivariable logistic regression model that adjusted GA variables to relevant clinical or treatment related factors. *P*-values < 0.05 were considered statistically significant. Statistical analyses were carried out using SPSS 28.0 for Windows (IBM, Armonk, NY, USA).

## Results

### Baseline patient characteristics

The study cohort consisted of 314 prostate cancer patients aged ≥ 65 years. Median age at diagnosis was 74 years (mean 73.5 ± 5.59 years). A total of 28 patients (8.9%) presented with low-risk prostate cancer, 139 patients (44.3%) with intermediate-risk cancer, and 147 patients (46.8%) with high-risk prostate cancer. Neoadjuvant ADT was administered in 294 patients (93.6%). Patient and treatment characteristics are summarized in Table 2.

**Table 2:** Patient and treatment characteristics

Parameter	Number of patients, *n* (%)
*Age*	
< 70	68 (21.7)
70–80	217 (69.1)
> 80	29 (9.2)
*Smoking status*	
Current	13 (4.1)
Former or never	300 (95.5)
Missing data	1 (0.3)
*Risk group*^*a*^	
High risk	147 (46.8)
Intermediate risk	139 (44.3)
Low risk	28 (8.9)
*Tumor stage*	
T1–T2	255 (81.2)
T3–T4	55 (17.5)
Missing data	4 (1.4)
*Lymph node involvement*	
Yes	7 (2.2)
No	306 (97.5)
Missing data	1 (0.3)
*Gleason score*	
≤ 6	49 (15.6)
7	149 (47.5)
8–10	116 (36.9)
*Initial PSA level*	
< 10	205 (65.3)
10–20	73 (23.2)
> 20	32 (10.2)
Median (mean ± SD)	8.2 (13.5 ± 24.3)
Missing data	4 (1.3)
*Neoadjuvant androgen deprivation*	
Yes	294 (93.6)
No	18 (5.7)
Missing data	2 (0.6)
*PSA before RT (ng/ml)*	
< 0.01	11 (3.5)
0.01–1.00	200 (63.7)
> 1.00	101 (32.2)
Median (mean ± SD)	0.45 (1.38 ± 2.96)
Missing data	2 (0.6)
*Radiation therapy*	
Conventional fractionation	301 (95.9)
Hypofractionation	13 (4.1)
*Pelvic lymph node irradiation*	
Yes	7 (2.2)
No	307 (97.8)
*Total dose*	
≤ 76 Gy	121 (38.5)
> 76 Gy	193 (61.5)
Median (mean ± SD)	78 Gy (76.3 ± 4.1)

*PSA* prostate-specific antigen, *SD* standard deviation, *RT* radiotherapy

^a^Risk group was defined according to National Comprehensive Cancer Network (NCCN) risk categories (Low: T1–T2a, GS ≤ 6 and PSA < 10, Intermediate: T2b–T2c and/or GS 7 and/or PSA 10–20, High: T3a or GS 8–10 or PSA > 20). The T stadium was estimated with the use of pretherapeutic magnetic resonance imaging (MRI), lymph node involvement was assessed with the use of MRI or computed tomography (CT) of the pelvis, supported by a PSMA PEC-CT.

### Results of comprehensive geriatric assessments

Comprehensive GA was performed in 282 patients; polypharmacy and comorbidities were recorded in 289 and 295 patients, respectively. The mean score on the Activities of Daily Living (ADL) scale was 99.04 ± 7.742; a score ≤ 90 indicating at least moderate dependency was detected in 6 patients (1.9%). The Instrumental Activities of Daily Living (IADL) scale had a mean score of 7.75 ± 0.874, and 18 patients (7.5%) had a score < 7, indicating decreased function. The mean time required for the Timed Up and Go test (TUG) was 10.54 ± 2.8 and 58 patients (18.5%) had reduced mobility as defined by a TUG > 10. The Mini Nutritional Assessment (MNA) scale had a mean value of 25.57 ± 1.825 and only 5 patients (1.6%) were at risk of malnutrition with an MNA score < 24.

The mean value on the Social Status and Support scale (SOS) was 23.05 ± 1.881 with a score ≤ 22 indicating a slightly higher need for social support detected in 82 patients (26.1%). Cognitive disorders defined as a score < 26 on the Mini-Mental State Examination (MMSE) were detected in 14 patients, a Geriatric Depression Scale (GDS) 15 score > 3 indicating a higher level of depression and anxiety was found in 57 patients (18.2%).

Polypharmacy defined as the intake of at least 6 medications was observed in 18.2% of patients, furthermore, the Charlson Comorbidity Index (CCI) revealed comorbidity in 31.5% of patients. The results of the geriatric assessment are displayed in Table 3.

**Table 3:** Results of the pretherapeutic geriatric assessment

Parameter	Number of patients, *n* (%)^a^
*ADL*	
≤ 90	6 (1.9)
> 90	276 (87.9)
Median (mean ± SD)	100 (99.04 ± 7.742)
*IADL*	
< 7	18 (7.5)
≥ 7	264 (84.1)
Median (mean ± SD)	8 (7.75 ± 0.874)
*TUG*	
≤ 10	218 (69.4)
> 10	58 (18.5)
Median (mean ± SD)	10 (10.54 ± 2.8)
*MNA*	
< 24	5 (1.6)
≥ 24	270 (86)
Median (mean ± SD)	28 (25.57 ± 1.825)
*SOS*	
≤ 22	82 (26.1)
> 22	199 (63.4)
Median (mean ± SD)	23 (23.05 ± 1.881)
*MMSE*	
< 26	14 (4.5)
≥ 26	269 (85.7)
Median (mean ± SD)	29 (28.43 ± 1.68)
*GDS*	
≤ 3	208 (66.2)
> 3	57 (18.2)
Median (mean ± SD)	1 (1.51 ± 2.078)
*CCI*	
0	190 (60.5)
≥ 1	99 (31.5)
Median (mean ± SD)	0 (0.41 ± 0.629)
*Polypharmacy (Number of medications)*	
≤ 6	238 (75.8)
> 6	57 (18.2)
Median (mean ± SD)	3 (3.73 ± 3.251)

*ADL* Activities of Daily Living, *IADL* Instrumental Activities of daily living, *TUG* Timed Up and Go Test, *MNA* Mini Nutritional Assessment, *SOS* Social status and support Survey, *MMSE* Mini-Mental State Examination, *GDS* Mini-Geriatric Depression Scale, *CCI* Charlson Comorbidity Index

^a^Comprehensive geriatric assessment was performed in 282 patients, polypharmacy and comorbidities were recorded in 289 and 295 patients

**Table 4:** Results of geriatric assessment among different age groups

Test	Age group	Minimum	Maximum	Median	Mean	SD	*P*-Value
ADL	< 70 Years 70–80 Years > 80 Years	65 30 95	100 100 100	100 100 100	99.29 99.38 99.79	4.566 5.409 1.021	0.772
IADL	< 70 Years 70–80 Years > 80 Years	2 4 3	8 8 8	8 8 8	7.65 7.83 7.38	1.194 0.648 1.313	*0.006*
TUG	< 70 Years 70–80 Years > 80 Years	5.2 5.5 7	18 24.4 19.28	9.50 10.12 11.44	9.89 10.67 11.92	2.99 2.690 3.077	*0.006*
MNA	< 70 Years 70–80 Years > 80 Years	21 23 22.5	30 30 30	27.5 28 28.25	27.29 27.77 27.52	1.60 1.32 2.07	0.163
SOS	< 70 Years 70–80 Years > 80 Years	18 16 15	25 25 25	23 24 23	23.16 23.15 22.58	1.462 1.607 2.339	0.522
MMSE	< 70 Years 70–80 Years > 80 Years	19 22 21	30 30 30	29 29 28.5	28.67 28.42 27.92	1.751 1.582 2.165	0.136
GDS	< 70 Years 70–80 Years > 80 Years	0 0 0	10 10 5	0 1 2	1.62 1.42 1.95	2.526 1.994 1.322	*0.026*
CCI	< 70 Years 70–80 Years > 80 Years	0 0 0	4 7 4	0 0 0	0.59 0.62 0.68	0.961 1.124 1.069	0.826
Polypharmacy	< 70 Years 70–80 Years > 80 Years	0 0 0	15 19 11	2 3 3	3.22 3.84 4.1	3.375 3.226 3.121	0.088

*ADL* Activities of Daily Living, *IADL* Instrumental Activities of daily living, *TUG* Timed Up and Go Test, *MNA* Mini Nutritional Assessment, *SOS* Social status and support Survey, *MMSE* Mini-Mental State Examination, *GDS* Mini-Geriatric Depression Scale, *CCI* Charlson Comorbidity Index, *SD* standard deviation

Numbers presented in *italic font* represent significant results

### Correlation between geriatric assessment results and age

We detected a significant association of the IADL score (*p* = 0.006) and the TUG result (*p* = 0.006) with age indicating a decreased function and mobility with increasing age. We also found a significant correlation between the GDS score and age (*p* = 0.026) suggesting a higher level of depression and anxiety among patients aged > 80 years. For the remaining geriatric assessment results, significant associations with age were not detected.

### Analysis of radiation-induced acute toxicity

Acute genitourinary and/or rectal toxicity grade ≥ 2 was detected in 40 patients (12.7%), acute genitourinary toxicity grade ≥ 2 was found in 37 patients (11.8%) and acute rectal toxicity grade ≥ 2 in 8 patients (2.5%), respectively. The association of baseline patient and treatment characteristics with acute radiation-induced toxicity is shown in Table 5.

**Table 5:** Univariable analysis of the association between patient and treatment characteristics and high-grade radiation-induced toxicity

	Association with acute rectal and/or genitourinary toxicity grade ≥ 2		Association with acute genitourinary toxicity grade ≥ 2		Association with acute rectal toxicity grade ≥ 2	
Parameter	OR (95%CI)	*P*-value	OR (95%CI)	*P*-value	OR (95%CI)	*P*-value
*Age (years)*						
< 70	1	–	1	–	1	–
70–80	2.100 (0.783–5.634)	0.141	2.468 (0.835–7.291)	0.102	0.938 (0.185–4.761)	0.939
> 80	2.016 (0.500–8.127)	0.324	2.560 (0.594–11.03)	0.207	n.a.	0.998
*Smoking status*						
Former/Never	1	–	–	–	–	–
Current	1.292 (0.276–6.061)	0.745	0.631 (0.080–5.001)	0.663	n.a.	0.999
*Risk group*						
Low risk	1	–	1	–	–	–
Intermediate risk	1.691 (0.366–7.807)	0.501	1.456 (0.312–6.796)	0.633	n.a	0.998
High risk	2.288 (0.506–10.34)	0.282	2.167 (0.478–9.814)	0.316	n.a	0.998
*Radiation therapy*						
*Conventional* fractionation	1	–	1	–	1	–
Hypofractionation	1.258 (0.269–5.897)	0.771	0.613 (0.077–4.859)	0.643	8.939 (1.617–49.41)	*0.012*
*Pelvic lymph node irradiation*						
No	1	–	1	–	1	–
Yes	2.908 (0.544–15.53)	0.202	1.290 (0.151–11.04)	0.816	7.119 (0.754–67.26)	0.087
*Total dose*						
≤ 76 Gy	1	–	1	–	1	–
> 76 Gy	1.351 (0.668–2.734)	0.402	1.557 (0.739–3.279)	0.244	0.619 (0.152–2.523)	0.503
*Neoadjuvant ADT*						
No	1	–	1	–	–	–
Yes	1.187 (0.263–5.370)	0.823	1.081 (0.238–4.902)	0.919	n.a.	0.999

*OR* odds ratio, *ADT* androgen deprivation therapy, *SD* standard deviation, *n.a.* not appliciable

Numbers presented in *italic font* represent significant results

### Association between geriatric parameters and radiation-induced acute toxicity

Univariable analysis showed a significant association between the presence of comorbidities and the development of acute rectal and/or genitourinary toxicity grade ≥ 2 (OR 2.269, 95%CI 1.130–4.557; *p* = 0.021). Regarding the relationship between GA variables and the development of acute genitourinary toxicity grade ≥ 2, a trend for increased toxicity in case of the presence of comorbidities was detected (OR 1.842, 95%CI 0.894–3.794; *p* = 0.098). Furthermore, univariable analysis revealed a significant relationship between the results on the SOS and the MMSE with rectal toxicity grade ≥ 2 (OR 0.156, 95%CI 0.030–0.823; *p* = 0.029 and OR 0.114, 95%CI 0.020–0.647; *p* = 0.014). A significant association between the intake of > 6 medications and the risk of rectal toxicity grade ≥ 2 was also detected (OR 4.415, 95%CI 1.070–18.222; *p* = 0.040). Furthermore, we observed a marginally significant association of reduced functionality represented by an ADL score ≤ 90 and the presence of comorbidities with acute rectal toxicity grade ≥ 2 (OR 0.111, 95%CI 0.011–1.102; *p* = 0.061 and OR 5.000, 95%CI 0.952–26.255; *p* = 0.057, respectively).

In multivariable analysis, GA results associated with toxicity grade ≥ 2 in univariable analysis (*p* < 0.1) were adjusted to variables deemed to be of clinical importance (age, smoking status, risk group, radiation fractionation, radiation dose, and pelvic node irradiation).

In multivariable analysis, the pretreatment CCI remained a significant predictor of acute genitourinary and/or rectal toxicity grade ≥ 2 (OR 2.633, 95%CI 1.260–5.502; *p* = 0.010), in addition, the CCI was significantly associated with acute genitourinary toxicity grade ≥ 2 (OR 2.169, 95%CI 1.017–4.625; *p* = 0.045) as well as with rectal toxicity grade ≥ 2 (OR 7.220, 95%CI 1.227–42.473; *p* = 0.029).

Furthermore, multivariable analysis revealed a significant association of the ADL score (OR 0.054, 95%CI 0.004–0.651; *p* = 0.022) with the risk of rectal toxicity grade ≥ 2. In addition, the score on the SOS scale (OR 0.159, 95%CI 0.028–0.891; *p* = 0.036) and polypharmacy (OR 4.618, 95%CI 1.045–20.405; *p* = 0.044) remained significant predictors of acute rectal toxicity grade ≥ 2 in multivariable analysis, whereas for the pretreatment MMSE, a marginally significant association with acute rectal toxicity grade ≥ 2 was detected (OR 2.144, 95%CI 0.019–1.083; *p* = 0.060). The results of uni- and multivariable analyses of the associations between geriatric parameters and radiation-induced acute toxicity are displayed in Table 6.

**Table 6:** Uni- and multivariable analysis of the association between pretherapeutic geriatric assessment (GA) scores and high-grade radiation-induced toxicity

	Association with acute rectal and/or genitourinary toxicity grade ≥ 2			Association with acute genitourinary toxicity grade ≥ 2			Association with acute rectal toxicity grade ≥ 2		
	Univariable analysis			Univariable analysis			Univariable analysis		
Parameter	OR (95%CI)	*P*-value	Adjusted *P*-value^a^	OR (95%CI)	*P*-value	Adjusted *P*-value^a^	OR (95%CI)	*P*-value	Adjusted *P*-value^a^
*ADL*	–	0.152	–	–	0.121	–	–	0.061	*0.022*
≤ 90	1			1			1		
> 90	0.281			0.253			0.111		
	(0.050–1.593)			(0.045–1.439)			(0.011–1.102)		
*IADL*	–	0.610	–	–	0.501	–	–	0.403	ultivariable analysis of the association between pret
< 7	1			1			1		
≥ 7	0.714			0. 641			0.395		
	(0.196–2.600)			(0.175–2.344)			(0.045–3.473)		
*TUG*	–	0.466	–	–	0.349	–	–	0.792	–
≤ 10	1			1			1		
> 10	1.356			1.485			0.747		
	(0.597–3.081)			(0.649–3.397)			(0.086–6.525)		
*MNA*	–	0.900	–	–	0.825	–	n.a.	0.999	–
< 24	1			1					
≥ 24	0.872			0.785					
	(0.102–7.464)			(0.091–6.736)					
*SOS*	–	0.136	–	–	0.143	–	–	*0.029*	*0.036*
≤ 22	1			1			1		
> 22	0.573			0.560			0.156		
	(0.276–1.191)			(0.263–1.196)			(0.030–0.823)		
*MMSE*	–	0.857	–	–	0.594	–	–	*0.014*	0.060
< 26	1			–			1		
≥ 26	0.868			1.755			0.114		
	(0.186–4.047)			(0.222–13.87)			(0.020–0.647)		
*GDS*	–	0.104	–	–	0.126	–	–	0.108	–
≤ 3	1			1			1		
> 3	1.923			1.895			3.796		
	(0.875–4.228)			(0.836–4.29)			(0.745–19.34)		
*CCI*	–	*0.021*	*0.010*	–	0.098	*0.045*	–	0.057	*0.029*
0	1			1			1		
≥ 1	2.269			1.842			5.000		
	(1.130–4.557)			(0.894–3.794)			(0.952–26.26)		
*Polypharmacy*	–	0.705	–	–	0.842	–	–	*0.040*	*0.044*
≤ 6	1			–			1		
> 6	1.177			1.094			4.415		
	(0.507–2.732)			(0.451–2.655)			(1.070–18.22)		

*ADL* Activities of Daily Living, *IADL* Instrumental Activities of daily living, *TUG* Timed Up and Go Test, *MNA* Mini Nutritional Assessment, *SOS* Social status and support Survey, *MMSE* Mini-Mental State Examination, *GDS* Mini-Geriatric Depression Scale, *CCI* Charlson Comorbidity Index, *OR* odds ratio, *95%CI* 95% confidence interval

^a^Parameters associated with toxicity grade ≥ 2 with a *p*-value < 0.1 in univariable analysis were adjusted to variables deemed to be of clinical importance (age, smoking status, risk group, radiation fractionation, radiation dose, and pelvic node irradiation)

## Discussion

Since chronological age alone is not an adequate indicator of the diverse aging process, a comprehensive assessment of older individuals’ medical, psychological, and functional abilities has become increasingly recognized as a means of distinguishing between those elderly patients who are good candidates for standard cancer treatment and those who are too vulnerable to tolerate aggressive therapies.

In this prospective observational study, we evaluated the usefulness of a comprehensive GA in predicting radiation-induced acute side effects in a group of prostate cancer patients over the age of 65 who received definitive radiotherapy. Our analysis revealed significant correlations between the incidence of acute radiation-induced toxicity and factors such as comorbidities, functional capacity, social standing, and polypharmacy.

In addition, we explored the occurrence of geriatric disorders across various age categories (< 70, 70–80, and > 80 years) and identified significant distinctions in measures of functional capacity (IADL and TUG) and mental state (GDS) across these age groups. These results imply that functionality tends to decrease and the likelihood of depression tends to increase with age. However, no significant differences between age groups were found in the other domains of geriatric assessment. These findings underscore the fact that age alone is inadequate for characterizing a patient’s medical, psychosocial, and functional abilities [27, 28].

However, it is well-established that geriatric patients are particularly vulnerable to toxicity due to age-related decline and heightened sensitivity to toxic exposures and it is essential to identify predictive factors for treatment response and toxicity.

Previous studies have primarily focused on the predictive role of a comprehensive GA for chemotherapy toxicity and have revealed that functional capacity and comorbidities were the factors most frequently linked with toxicity [11, 12, 29, 30]. For instance, in a study by Hurria et al. that analyzed risk factors for the toxicity of chemotherapy in geriatric cancer patients aged 65–91 years, a need for assistance in activities of daily living such as mobility, housework, and medication intake and also reduced mobility in walking test correlated with treatment-related side effects [31]. In addition, a systematic review and meta-analysis of 13 studies by Edwards et al. revealed that patients with comorbidities had a higher risk of experiencing severe chemotherapy-induced toxicity than those without comorbidities [32]. Furthermore, cognitive impairment, social dependency, and depression have been found to represent predictors of chemotherapy-related side effects [12, 33–35]. Previous studies have also developed predictive models for chemotherapy toxicity in older adults with cancer that incorporate geriatric assessment variables. For example, the Chemotherapy Risk Assessment Scale for High-Age Patients (CRASH) and the Cancer Aging Research Group (CARG) toxicity score have both been shown to represent reliable predictors of chemotherapy toxicity and overall survival in older cancer patients [8, 12, 26, 36].

To date, evidence is scarce on what type of GA tools or predictors of health status can be employed to anticipate the risks of radical prostatectomy in older males. Previous studies that have investigated the relationship between frailty and complication rates among patients undergoing radical prostatectomy have revealed an increased risk of overall and major complications in frail patients [37, 38]. Patients with elevated frailty scores are prone to experience higher rates of wound disruptions, bleeding transfusions, and 30-day mortality [34]. Nevertheless, further research is necessary to ascertain the specific clinical tools that can directly predict the surgical tolerability of elderly men.

In the present study, the pretreatment CCI was significantly associated with the risk of high-grade rectal and genitourinary side effects. Moreover, our findings revealed that ADL, social status, and polypharmacy were significantly linked to rectal toxicity, and we also observed a trend suggesting a higher risk of rectal toxicity among patients with cognitive impairment.

The results of our study can be attributed to the assumption that the presence of comorbidities indicates inflammatory processes or microvascular alterations that negatively influence the recuperative capacity of healthy tissues and result in heightened toxicity. Additionally, the observed trend towards a heightened risk of rectal side effects among patients with cognitive impairments supports to the hypothesis of a link with vascular changes, which are also prevalent in individuals with dementia. ADL and social status serve as measures of individual independence in performing daily tasks. For patients with difficulties in performing basic activities and those with unfulfilled social requirements, it might be challenging to adhere to recommended diets and other supportive measures. This group of patients may also subjectively experience a greater degree of discomfort from gastrointestinal symptoms compared to those who do not face similar limitations in their daily lives. Polypharmacy is a widely acknowledged risk factor for heightened toxicity among older patients receiving cancer treatment [39]. The consumption of numerous medications may heighten the susceptibility of healthy tissues to radiation exposure; moreover, certain drugs are known to cause gastrointestinal reactions, which, when coupled with an additional irritating factor, such as ionizing radiation, can lead to the manifestation of drug-related side effects that were not clinically apparent prior to the radiotherapy.

To date, data on the role of a CGA for the prediction of radiation-induced toxicity are sparse. Goineau et al. aimed to identify predictors of impaired quality of life (QoL) in men aged 75 years or older who underwent curative radiotherapy with or without androgen deprivation therapy (ADT) for localized prostate cancer. Comprehensive GA and QoL questionnaires were administered to 208 elderly prostate cancer patients. However, none of the parameters studied, including tumor characteristics, treatment, or oncogeriatric parameters, were predictive of a decrease in QoL following radiotherapy [40]. DeVries et al. investigated whether GA items or frailty screening instruments could predict the likelihood of acute toxicity in 160 head and neck cancer patients undergoing radiation therapy and also did not find any significant association between frailty or geriatric parameters and the risk of acute toxicity [22]. In contrast, Ulger et al. detected a significant correlation between lower gait speed and emesis when evaluating the predictive value of a comprehensive GA for radiation therapy toxicity and tolerability in 30 geriatric cancer patients with a mean age of 70 years [41].

In the present prospective study, we performed a comprehensive and systematic analysis of the predictive role of geriatric parameters. To the best of our knowledge, our study cohort is the largest to date that has examined the correlation between the GA and the risk of radiation-induced toxicity. It is also characterized by a high degree of homogeneity in terms of patient, tumor, and treatment characteristics. In addition, we focused on the predictive value of the CGA for grade ≥ 2 side effects, which are regarded as clinically significant adverse events.

The integration of a comprehensive GA in treatment planning and follow-up has been associated with improved overall survival, quality of life, and physical function [42–44]. In prostate cancer radiotherapy, the pretherapeutic GA may assist in determining the appropriate fractionation schedule or target volume. For instance, in patients who are particularly vulnerable, the GA might indicate the omission of elective lymph node irradiation or avoiding fractionation schedules that are known to result in higher toxicity rates.

A potential drawback of our study is a potential selection bias, whereby certain vulnerable patients who were deemed eligible for radiation therapy may have declined to participate due to their overall diminished performance status. Moreover, unaccounted factors such as prostate volume and drug treatment such as alpha-blocker administration may have influenced the occurrence of side effects. Furthermore, in patients undergoing hypofractionated radiotherapy, acute side effects are frequently observed following treatment completion, so that possibly acute side effects were not fully captured in this subgroup of patients. In addition, detailed analyses of dose–volume parameters were beyond the scope of the present study. However, due to our strict dose constraints, we were able to achieve a high level of homogeneity regarding the dose–volume parameters in our study population. It has also to be taken into account that a dose–volume histogram of the bladder or rectum obtained from a single planning computed tomography is unlikely to represent the true dose distribution delivered to the bladder or rectum during the treatment course.

Nevertheless, validation of our data in additional prospective large-scale studies is imperative before firm conclusions about the effectiveness of geriatric assessment in predicting radiation-induced toxicity in prostate cancer can be drawn.

In conclusion, the significant correlation we observed between comorbidities, functionality, social status, and polypharmacy and the risk of developing acute radiation-induced side effects supports the hypothesis that the GA may serve as a useful tool in predicting radiotherapy toxicity in elderly cancer patients. If confirmed by additional studies, the GA could contribute to the identification of patients at high risk of adverse events, leading to individualized treatment plans that can minimize toxicity in elderly cancer patients.
